# Activation of glutamine synthetase (GS) as a new strategy for the treatment of major depressive disorder and other GS-related diseases

**DOI:** 10.1038/s41401-024-01441-2

**Published:** 2025-01-07

**Authors:** Jae Soon Kang, Hwajin Kim, Ji Hyeong Baek, Miyoung Song, Hyeongchan Park, Wonjune Jeong, Hye Jin Chung, Dae Young Yoo, Dong Kun Lee, Sang Won Park, Hyun Joon Kim

**Affiliations:** 1 Department of Anatomy and Convergence Medical Science, College of Medicine, Institute of Medical Science, Tyrosine Peptide Multiuse Research Group, Anti-aging Bio Cell Factory Regional Leading Research Center, Gyeongsang National University, Jinju, Gyeongnam Republic of Korea; 2 Department of Pharmacology and Convergence Medical Science, College of Medicine, Institute of Medical Science, Tyrosine Peptide Multiuse Research Group, Anti-aging Bio Cell Factory Regional Leading Research Center, Gyeongsang National University, Jinju, Gyeongnam Republic of Korea; 3 College of Pharmacy and Research Institute of Pharmaceutical Sciences, Gyeongsang National University, Jinju, Gyeongnam Republic of Korea; 4 Department of Anatomy and Cell Biology, College of Veterinary Medicine, Research Institute for Veterinary Science, Seoul National University, Seoul, Republic of Korea; 5 Department of Physiology and Convergence Medical Sciences, College of Medicine, Institute of Medical Science, Tyrosine Peptide Multiuse Research Group, Gyeongsang National University, Jinju, Gyeongnam Republic of Korea

**Keywords:** depression, glutamine synthetase, tyrosine nitration, tyrosine-containing dipeptide, epilepsy, hyperammonemia

## Abstract

Glutamine synthetase (GS) plays a crucial role in the homeostasis of the glutamate–glutamine cycle in the brain. Hypoactive GS causes depressive behaviors. Under chronic stress, GS has no change in expression, but its activity is decreased due to nitration of tyrosine (Tyr). Thus, we speculate that agents that prevent nitration or facilitate denitration of GS would be candidates for new antidepressants. Using human recombinant GS and mouse lysate from the medial prefrontal cortex, we demonstrated that Tyr (0.0313−0.5 µM) dose-dependently protected GS activity against peroxynitrite-induced Tyr-nitration of GS. Diet supplementation with Tyr exerted significant antidepressant effects in a chronic immobilization stress depression mouse model. We further found that dipeptides, such as tyrosyl-glutamine (YQ), that had appropriate chemical properties for medication also increased GS activity both in vitro and in vivo and exerted antidepressant effects. Because reduced GS activity also occurs in epilepsy and hyperammonemia, we evaluated whether Tyr and YQ had therapeutic effects. Interestingly, Tyr or YQ administration significantly attenuated kainic acid-induced seizures in mice and reduced blood ammonia levels in azoxymethane- or bile duct ligation-induced hyperammonemia mouse models, which was accompanied by an increment in GS activity. The activation of GS was accomplished by a decrement in Tyr-nitration, so-called Tyr-denitration. Therefore, this study demonstrates that the activation of GS could be a new strategy to treat depression and other GS-related diseases.

## Introduction

Major depressive disorder (MDD) is the first leading disease of disability worldwide [1]. Many people who suffer from depressive episodes with or without a diagnosis of MDD sometimes feel a strong suicidal impulse [2]. During the COVID-19 pandemic, a tremendous increase in anxiety and depression occurred among people in young age groups [3]. Unfortunately, no medications without side effects are available. Therefore, it is very urgent and important to develop effective and safe medications that treat symptoms of depression, particularly those that could worsen with stressful events and/or chronic stress.

Contemporary humans cannot avoid stressful life events and thus experience both large and small stressors in daily life. Chronic stress can evoke a variety of diseases from the common cold to cancer [4–8]. MDD is also widely accepted as a disease that is mainly induced by chronic stress [9]. Thus, animal models of MDD have been developed using chronic stress regimens to investigate the underlying mechanisms and develop medications to treat [10, 11]. In our laboratory, a chronic immobilization stress (CIS)–induced mouse model of depression was established [12–14] and an unbalanced glutamate (Glu)-glutamine (Gln) cycle was found in the medial prefrontal cortex (mPFC) of depressed mice [15, 16]. The unbalanced Glu–Gln cycle resulted in disrupted homeostasis of the levels of Glu and Gln, which has been found in human MDD patients [17, 18]. Glutamine synthetase (GS) plays a crucial role in homeostatic maintenance of the Glu–Gln cycle by providing Gln as a substrate of Glu for glutamatergic neurotransmission [19]. Experimental ablation or inhibition of GS decreased Glu and Gln levels in the mPFC, which resulted in depressive behaviors with hypoactive glutamatergic neurotransmission [15, 16]. Thus, it is strongly suggested that normal GS activity is crucial to maintain glutamatergic activity. Moreover, we found that depressive behaviors are directly affected by neurotransmission of glutamatergic neurons in the mPFC [16]. These findings have been reinforced by other reports [20–24]. Esketamine and dextromethorphan-bupropion were recently approved for clinical use as antidepressants by the United States Food and Drug Administration, and the working mechanism is based on their rapid increase in prefrontal glutamatergic signaling [25, 26]. These findings support our hypothesis that glutamatergic activation might be a suitable target for MDD treatment.

Previously, we revealed that CIS decreases GS activity in the mPFC, which causes depressive behaviors [16]. The lower activity or expression of GS were frequently found in postmortem brain of MDD patients [27]. Regulation of GS activity may occur via several posttranslational modifications including tyrosine (Tyr)-nitration by peroxynitrite (PN), which is produced by hydrogen peroxide and nitric oxide [28, 29]. Tyr-nitration is also accepted as a natural inhibitory mechanism of GS in a variety of organisms due to increments in reactive oxygen and nitrogen species (ROS/RNS). Thus, Tyr-nitration is considered a biomarker of nitroxidative stress [30]. Chronic stress is a main cause of ROS/RNS elevation in the brain and in other organs [31, 32]. Thus, we speculate that Tyr-nitration of GS is a main cause of decreased activity in the CIS-induced depressive mouse mPFC because no change occurred in GS expression [16]. Therefore, in the present study, we first examined whether the GS activity decrease is due to the increment of Tyr-nitration. Interestingly, we found that Tyr-nitration of GS was increased in the brain by CIS. This finding suggested the hypothesis that a drug that increased GS activity by preventing nitration or facilitating denitration would be an effective antidepressant. Moreover, many other studies have reported that incremented Tyr-nitration of GS is also found in other diseases and conditions including seizures and hyperammonemia [28, 33, 34]. Therefore, we evaluated whether the same strategy, GS activation, would be effective in seizure and hyperammonemia mouse models, as well as in a CIS-induced depression mouse model, as a potential therapeutic strategy.

## Materials and methods

### Animals

Male C57BL/6 and ICR mice were purchased from Koatech (Seoul, Rep. of Korea). All animals were cared for in the animal facility at Gyeongsang National University (GNU). Mice were housed in cages in a specific pathogen-free room at 24 ± 2 °C and 60% ± 10% humidity. The light-dark cycle was 12:12 h. Diets and water were provided *ad libitum*. Mice were treated in accordance with the National Institutes of Health (Bethesda, MD, USA) guidelines and protocols approved by the GNU Institutional Animal Care and Use Committee (summarized in Table S1).

### Tyr and Tyr-containing peptide diets

Tyr was purchased from Nutricost (Vineyard, UT, USA), and tyrosyl-glutamine (YQ)/glutamyl-tyrosine (QY) was synthesized at 95% purity (CSBio, Menlo Park, CA, USA). To deliver Tyr, YQ, and QY to mice, calorie-balanced diets containing Tyr (Y), YQ, or QY were prepared (Raonbio, Yongin, Rep. of Korea), which included 181.19 mg/kg (1×), 543.57 mg/kg (3×), or 905.95 mg/kg (5×) Y or 309.32 mg/kg (1×) or 927.96 mg/kg (3×) YQ/QY.

### Preparation of animal models of disease

The animal disease models for depression, seizure, and acute liver injury were induced as described in previous studies, and the method details are explained in the supplemental information.

### Behavior tests

Depressive behavior tests, including the open field test (OFT), elevated plus maze (EPM), tail suspension test (TST), forced swimming test (FST), and sucrose preference test (SPT) were conducted as previously described [12, 15].

### Preparation of plasma and tissue lysate

Blood and tissue were collected from mice after decapitation under anesthesia induced by CO_2_. The plasma was isolated from blood, mixed with EDTA to prevent clotting, and centrifuged at 1500 × *g* for 10 min at 4 °C. Tissues were immediately snap-frozen in liquid nitrogen for storage at −80 °C for lysis or fixed in 10% buffered formalin for perfusion. After weighing the tissue, it was immersed in an appropriate volume of ice-cold radioimmunoprecipitation assay buffer with a complete protease inhibitor cocktail (04693116001, Roche, Basel, Switzerland). The tissue was crushed using a Bullet Blender Tissue Homogenizer (Next Advance, Troy, NY, USA) and sequentially sonicated for complete homogenization. The lysate was separated by centrifugation at 12,000 × *g* for 20 min at 4 °C. After the protein assay, the plasma, and lysate were stored at −80 °C before use.

### Biochemical and molecular approaches

Measurements of ammonia, alanine aminotransferase (ALT), alkaline phosphatase, corticosterone (CORT), and ROS/RNS, GS activity assays, and Western blotting were conducted to evaluate the therapeutic effects of Y and Y-containing dipeptides. The method details are provided in the supplemental information.

### In vitro denitration tests

A total of 10 µg of mPFC lysate and 0.5 µg of active recombinant human GS (rhGS, NBP2-52619, Novus Biologicals, Centennial, CO, USA) were mixed with PN (516620, Calbiochem, San Diego, CA, USA) in serially diluted Y, YQ, or QY and then placed on ice for 5 min. GS activity assay was performed as described in the supplemental information.

### Measurement of spontaneous excitatory postsynaptic currents

To measure electrophysiological changes, 8-week-old male Vglute2-Cre::CRISPR-CAS9 mice were chronically stressed by immobilization for 2 h daily for 15 days. After CIS, brain slice preparation and recording of spontaneous excitatory postsynaptic currents (sEPSCs) were conducted as previously described [16]. Briefly, sEPSCs were isolated by blocking γ-aminobutyric acid (GABA)_A_ receptors with 100 μM picrotoxin (P1675, Merck, Rahway, NJ, USA), and electrophysiological signals were low-pass filtered at 2–5 kHz. The signals were analyzed using Clampfit version 11.1 (Molecular Devices, CA, USA) and MiniAnalysis version 6 (Synapto soft, NJ, USA).

### Amino acid analysis

Glu, Gln, and GABA levels in the plasma and mPFC were quantified without derivatization using liquid chromatography with tandem mass spectrometry as previously described [16]. Amino acids were quantified using the multiple reaction monitoring detection method under the following conditions: *m*/*z* 148 to 84, *m*/*z* 147 to 84, *m*/*z* 104 to 87, and *m*/*z* 153 to 88 for Glu, Gln, GABA, and the internal standard, respectively.

### Statistical analysis

All data are shown as the mean ± SEM and were statistically analyzed using one-way analysis of variance with a *post hoc* Dunnett’s multiple comparisons test or two-tailed/unpaired Student’s *t*-test (*P* < 0.05) using GraphPad Prism 9 (GraphPad Software, La Jolla, CA, USA).

## Results

### Chronic stress decreases GS activity through Tyr-nitration in the mPFC

**Fig. 1:**
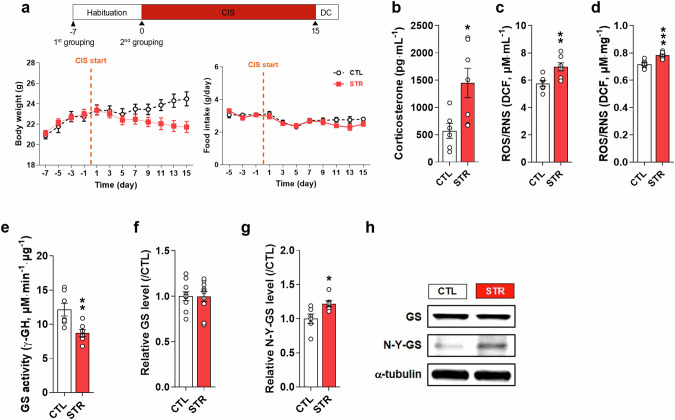
Tyrosine nitration (N-Y) of glutamine synthetase (GS) is increased in the medial prefrontal cortex (mPFC) in the stress group (STR) compared with the control group (CTL) by chronic immobilization stress (CIS). **a** Experimental scheme for CIS (upper panel). Changes in body weight (lower left) and food intake (lower right) during the experiment. **b** Plasma corticosterone level. **c**, **d** Reactive oxygen species (ROS)/reactive nitrogen species (RNS) levels in plasma and the mPFC, respectively. **e**, **f** Activity and expression levels of GS. **g** nitrotyrosine-GS level in the mPFC. **h** Representative Western blot images for GS, N-Y-GS, and α-tubulin. Data are presented as the mean±SEM. *P < 0.05, **P < 0.01, ***P < 0.001 (*t*-test).

In CIS-induced depression mice (stressed, STR, Fig. 1a), CORT and ROS/RNS were increased in the plasma (Fig. 1b and c; *t* = 2.773, df = 11, *P* = 0.0181 and *t* = 3.163, df = 11, *P* = 0.009). There was also an increase in ROS/RNS (Fig. 1d, *t* = 4.861, df = 12, *P* < 0.001) and a decrease in GS activity (*t* = 3.273, df = 12, *P* = 0.007) without a change in GS expression in the mPFC in the STR group compared with those in the control (CTL) group (Fig. 1e and f). An immunoprecipitation study showed greater Tyr-nitration of GS in the STR group than in the CTL group (Fig. 1g and h, *t* = 2.611, df = 10, *P* = 0.0260).

### Tyr has antidepressive and anti-anxiety properties

**Fig. 2:**
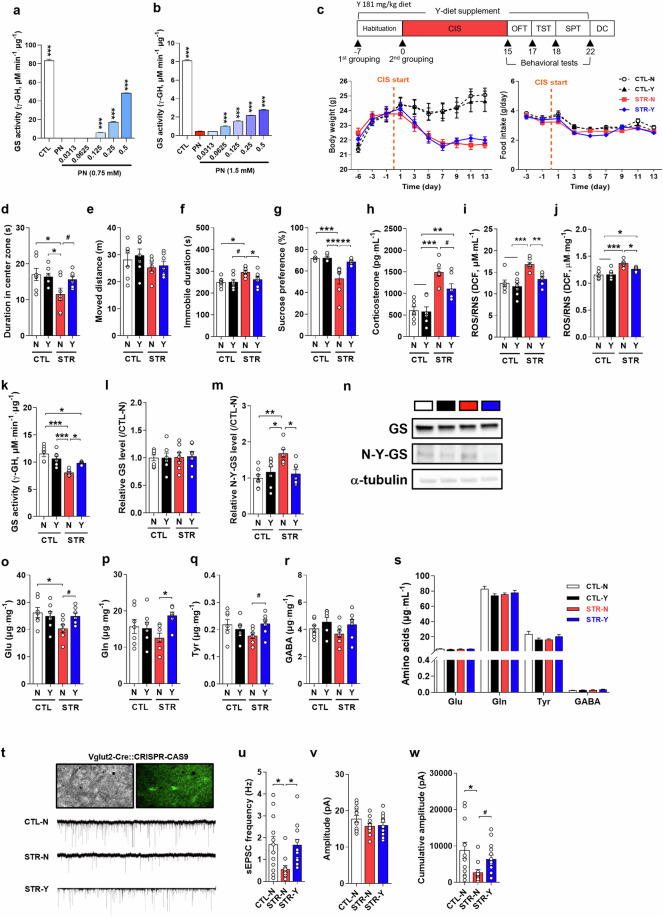
Tyrosine (Y) shows antidepressive effects in chronic immobilization stress (CIS)-induced depressive mice via activation of glutamine synthetase (GS) in the medial prefrontal cortex (mPFC). **a** and **b** Denitrative effect of Y on human recombinant GS and mouse mPFC lysate. **c** Scheme for Y diet supplementation, CIS, and behavioral tests. Changes in body weight and food intake during the experiment among groups (normal diet control group: CTL-N, Y diet control group: CTL-Y, normal diet stress group: STR-N, Y diet stress group: STR-Y, *n* = 7 per group). **d**–**g** Behavioral test results: open field test (**d** and **e**), tail suspension test (**f**), and sucrose preference test (**g**). **h** Plasma corticosterone level. **i** and **j** Reactive oxygen species (ROS)/reactive nitrogen species (RNS) levels in plasma and the mPFC, respectively. **k** and **l** Activity and expression levels of GS. **m** Nitrotyrosine-GS (N-Y-GS) level in the mPFC. **n** Representative Western blot images for GS, N-Y-GS, and α-tubulin. **o**–**r** Glutamate (Glu), glutamine (Gln), Tyr, and γ-aminobutyric acid (GABA) levels in the mPFC. **s** Amino acid (Glu, Gln, Tyr, and GABA) levels in plasma. **t** Traces of spontaneous excitatory postsynaptic currents (sEPSCs) in glutamatergic neurons of the mPFC. The changes of frequency (**u**), amplitude (**v**), and cumulative amplitude (**w**) among groups. Data are presented as the mean ± SEM. *P < 0.05, **P < 0.01, ***P < 0.001 (multiple comparisons test), and ^#^*P* < 0.05 (individual comparison test) vs. CTL-N or STR-N groups.

We examined the denitration of GS via Tyr using rhGS and mouse mPFC lysate (Fig. 2a and b). Tyr protected GS activity against PN-induced Tyr-nitration of GS in a dose-dependent manner. Mice were subjected to CIS and behavior tests with or without a Tyr-supplemented diet (Fig. 2c). A bodyweight difference was only found between the CTL and STR groups, and no difference was found between the normal diet (N) and Tyr-supplemented diet groups. Interestingly, mice fed a Tyr-supplemented diet (STR-Y mice) showed fewer depressive symptoms including anxiety-related behaviors, helplessness, or anhedonic behaviors than did STR-N mice (Fig. 2d–g; d, *F*_(3,24)_ = 3.446, *P* = 0.033; *t* = 2.190, df = 12, *P* = 0.0490; f, *F*_(3,24)_ = 4.224, *P* = 0.016; g, *F*_(3,24)_ = 11.11, *P* < 0.001). Plasma CORT and ROS/RNS levels were significantly decreased in STR-Y mice compared with those in STR-N mice (Fig. 2h and i; h, *F*_(3,24)_ = 16.49, *P* < 0.001; i, *F*_(3,24)_ = 11.26, *P* < 0.001), and ROS/RNS levels in the PFC were also decreased in STR-Y mice compared with those in STR-N mice (Fig. 2j, *F*_(3,24)_ = 14.65, *P* < 0.001). The activity, expression, and Tyr-nitration of GS were analyzed simultaneously. Reduced GS activity was increased by Tyr treatment (*F*_(3,24)_ = 15.27, *P* < 0.001) with no change in GS expression (Fig. 2k and l). Tyr-nitration of GS was increased in STR mice compared with that in CTL mice but was decreased by Tyr supplementation (*F*_(3,24)_ = 6.482, *P* = 0.0023) (Fig. 2m and n). Because GS activity directly affects Glu, Gln, and GABA in the mPFC, we analyzed their amounts in both the PFC and plasma (Fig. 2o–s). CIS decreased Glu and Gln in the mPFC (Glu, *F*_(3,24)_ = 2.696, *P* = 0.0685; *t* = 2.463, df = 12, *P* = 0.0299; Gln, *F*_(3,24)_ = 2.689, *P* = 0.0690) but did not affect their plasma levels. Tyr supplementation reversed Glu, Gln, and Tyr, but not GABA, to CTL levels. To evaluate the recovery of glutamatergic signaling with Tyr supplementation, sEPSCs were measured using glutamatergic neuron-specific labeled transgenic mice (Vglut2-Cre::CRISPR-CAS9) (Fig. 2t). We found that the antidepressive and anti-anxiety effect of Tyr was closely related to the increment in glutamatergic neurotransmission (Fig. 2t–w; u, *F*_(2,33)_ = 5.048, *P* = 0.0122; w, *F*_(2,33)_ = 4.307, *P* = 0.0213; *t* = 2.686, df = 24, *P* = 0.0129).

### Tyr-Gln (YQ) and Gln-Tyr (QY) have antidepressive and denitration effects on GS

**Fig. 3:**
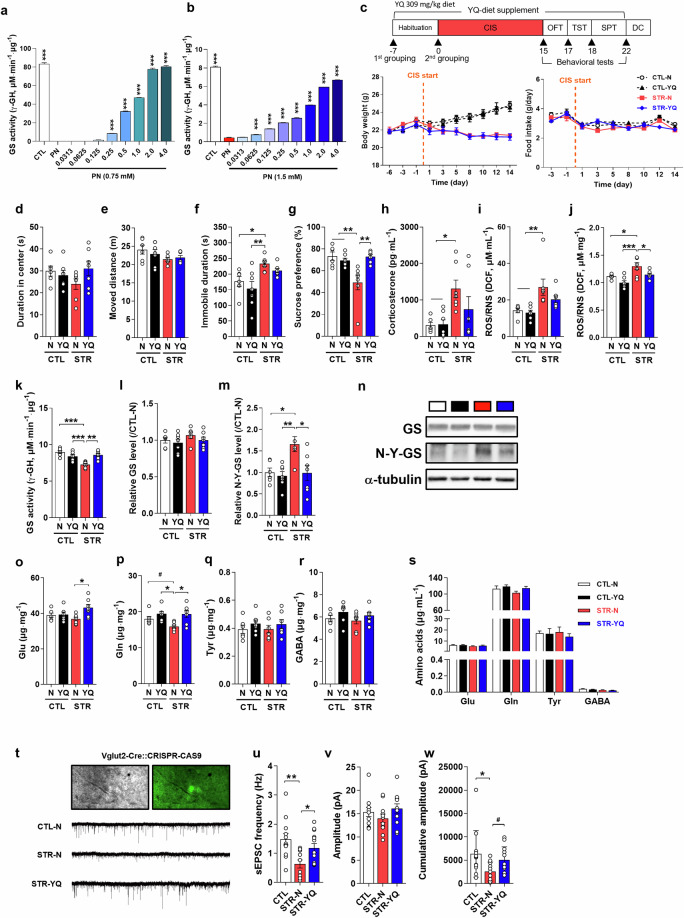
Tyr-Gln (YQ) shows antidepressive effects in chronic immobilization stress (CIS)-induced depressive mice via activation of glutamine synthetase (GS) in the medial prefrontal cortex (mPFC). **a** and **b** Denitrative effect of YQ on human recombinant GS and mouse mPFC lysate. **c** Scheme for YQ diet supplementation, CIS, and behavioral tests. Changes in body weight and food intake during the experiment among groups (normal diet control group: CTL-N, YQ diet control group: CTL-YQ, normal diet stress group: STR-N, YQ diet stress group: STR-YQ, *n* = 7 per group). **d**–**g** Behavioral test results: open field test (**d** and **e**), tail suspension test (**f**), and sucrose preference test (**g**). **h** Plasma corticosterone level. **i** and **j** Reactive oxygen species (ROS)/reactive nitrogen species (RNS) levels in plasma and the mPFC, respectively. **k** and **l** Activity and expression levels of GS. **m** Nitrotyrosine-GS (N-Y-GS) level in the mPFC. **n** Representative Western blot images for GS, N-Y-GS, and α-tubulin. **o**–**r** Glutamate (Glu), glutamine (Gln), Tyr, and γ-aminobutyric acid (GABA) levels in the mPFC. **s** Amino acid (Glu, Gln, Tyr, and GABA) levels in plasma. **t** Traces of spontaneous excitatory postsynaptic currents (sEPSCs) in glutamatergic neurons of the mPFC. Changes in frequency (**u**), amplitude (**v**), and cumulative amplitude (**w**) among groups. Data are presented as the mean ± SEM. *P < 0.05, **P < 0.01, ***P < 0.001 (multiple comparisons test), and ^#^*P* < 0.05 (individual comparison test) vs. CTL-N or STR-N groups.

To examine the bioavailability of YQ and QY, we administered mice to each dipeptide-supplemented diet and examined whether the dipeptides had an antidepressive effect in a CIS-induced depression model (Figs. 3 and S1). Before the in vivo experiment, we performed an in vitro test of the denitration effect of the dipeptides on GS compared with that on PN using rhGS and mouse brain lysate (Figs. 3a–b and S1a–b), similar to our approach with Tyr. We found that a higher concentration could be applied and more denitration of GS was achieved, indicating the expanded clinical availability of dipeptides compared with that of the single amino acid Tyr. When YQ- or QY-supplemented diets were tested in our well-established CIS regimen, YQ showed antidepressive effects in behavioral tests (Fig. 3d–g; g, *F*_(3,23)_ = 7.926, *P* < 0.001) and QY showed similar mean differences in each behavior test (Fig. S1d–g; d, *F*_(3,25)_ = 7.864, *P* < 0.001; f, *F*_(3,25)_ = 4.430, *P* = 0.013; g, *F*_(3,25)_ = 3.560, *P* = 0.029). The CORT levels in plasma and ROS/RNS levels in both plasma and the mPFC were also reduced by YQ or QY treatment (Figs. 3h–j and S1h–j; h, *F*_(3,23)_ = 4.138, *P* = 0.0175; i, *F*_(3,23)_ = 5.756, *P* < 0.001; j, *F*_(3,23)_ = 9.056, *P* < 0.001; Fig. S1h, *F*_(3,25)_ = 11.06, *P* < 0.001; S1i, *F*_(3,25)_ = 8.375, *P* < 0.001; S1j, *F*_(3,25)_ = 14.15, *P* < 0.001). As expected, YQ or QY increased GS activity compared with that in the STR-N group without GS expression changes (Figs. 3k and l,  S1k and l; Fig. 3k, *F*_(3,23)_ = 13.64, *P* < 0.001; Fig. S1k, *F*_(3,25)_ = 10.95, *P* < 0.001). An increase in the Tyr-nitration of GS was observed in STR-N mice, but both YQ and QY remarkably reduced the Tyr-nitration of GS to the level observed in CTL-N mice (Figs. 3m and n, S1m and n; Fig. 3m, *F*_(3,23)_ = 5.803, *P* = 0.004; Fig. S1m, *F*_(3,25)_ = 6.055, *P* = 0.003). Tyr-Gln increased Glu and Gln levels in the mPFC (Fig. 3o and p; o, *F*_(3,23)_ = 3.573, *P* = 0.029, p, *F*_(3,23)_ = 5.093, *P* = 0.008), but QY did not affect these amino acid levels (Fig. S1o and p). No changes were found in plasma Glu, Gln, Tyr, or GABA levels, similar to the effects observed after Tyr treatment (Fig. 2s). Although CIS evoked hypoactive glutamatergic neurotransmission, YQ recovered sEPSCs and the cumulative amplitude to levels observed in CTL mice (Fig. 3t–w; u, *F*_(2,33)_ = 5.048, *P* = 0.0122; w, *F*_(2,33)_ = 4.307, *P* = 0.0213; *t* = 2.686, df = 24, *P* = 0.0129).

### Both pre- and post-supplementation with YQ produce antidepressive and denitration effects

**Fig. 4:**
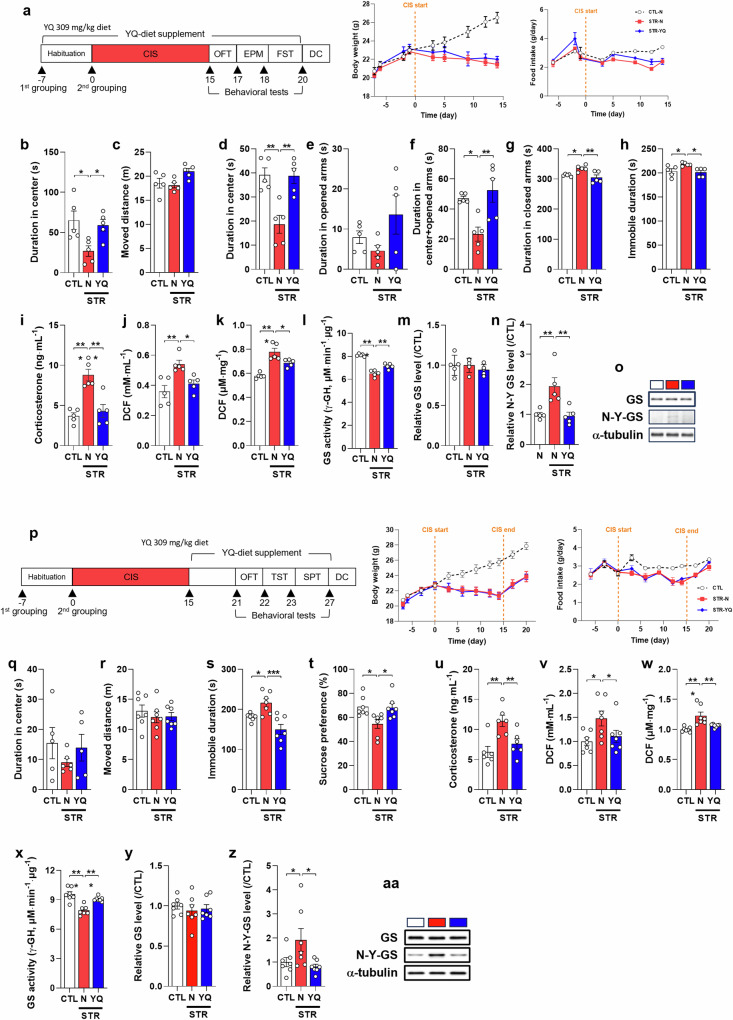
Tyr-Gln (YQ) of both pre- (a-o) and post-supplementation (p-aa) shows antidepressive effects in chronic immobilization stress (CIS)-induced depressive mice. **a** and **p** Experiment scheme of YQ supplementation including CIS and behavior tests and change of body weight and daily food intake during experiment (normal diet control group: CTL, normal diet stress group: STR-N, YQ diet stress group: STR-YQ, *n* = 5 for pre-supplementation and *n* = 7 for post-supplementation per group). **b**, **c**, and **q**, **r** Open field test. **d**–**g** Elevated plus maze. **h** Forced swimming test. **i** and **u** Corticosterone level in plasma. **j** and **v** Reactive oxygen species (ROS)/reactive nitrogen species (RNS) level in plasma. **k** and **w** ROS/RNS level in mPFC. **l** and **x** Activity of glutamine synthetase (GS) in mPFC. **m** and **y** Relative GS expression level in mPFC. **n** and **z** Relative nitrotyrosine-GS (N-Y-GS) level in mPFC. **o** and **aa** Representative Western blot images for GS, N-Y-GS, and α-tubulin. **s** Tail suspension test. **t** Sucrose preference test. Data are presented as the mean ± SEM. *P < 0.05, **P < 0.01, ***P < 0.001 (multiple comparisons test) vs. CTL or STR-N groups.

To evaluate whether YQ could be used to prevent depression and treat depression after onset, we provided a diet containing YQ before and after CIS (Fig. 4a and p). Both pre- and post-supplementation of YQ showed antidepressive effects on CIS-induced depressive behaviors. The STR-YQ group, which was pre-supplied with YQ, showed an increased duration in the center in the OFT, an increased duration in the open arms + center in the EPM, and a decreased duration in the closed arms compared with those in the STR-N group (Fig. 4b–g; b, *F*_(2,12)_ = 5.631, *P* = 0.019; d, *F*_(2,12)_ = 12.86, *P* = 0.001; f, *F*_(2,12)_ = 8.438, *P* = 0.005; g, *F*_(2,12)_ = 7.868, *P* = 0.007). In the FST, the STR-YQ group also showed a decrease in immobile duration compared with that in the STR-N group (Fig. 4h; *F*_(2,12)_ = 5.355, *P* = 0.022). The CORT and ROS/RNS levels in the plasma and mPFC were lower in the STR-YQ group than those in the STR-N group (Fig. 4i–k; i, *F*_(2,12)_ = 17.29, *P* < 0.001; j, *F*_(2,12)_ = 9.177, *P* = 0.004; k, *F*_(2,12)_ = 23.76, *P* < 0.001). GS activity was restored in the STR-YQ group without a change in the GS expression level (Fig. 4l and m; l, *F*_(2,12)_ = 67.08, *P* < 0.001), and Tyr-nitration of GS was decreased in the STR-YQ group compared with that in the STR-N group (Fig. 4n and o; n, *F*_(2,12)_ = 9.303, *P* = 0.004).

The STR-YQ group, which was post-supplied with YQ, showed a decreased immobile duration in the TST and an increased sucrose preference compared with those in the STR-N group (Fig. 4s–t; s, *F*_(2,18)_ = 11.95, *P* < 0.001; t, *F*_(2,18)_ = 5.676, *P* = 0.012). The CORT and ROS/RNS levels in the plasma and mPFC were lower in the STR-YQ group than those in the STR-N group (Fig. 4u–w; u, *F*_(2,18)_ = 8.673, *P* = 0.003; v, *F*_(2,18)_ = 4.082, *P* = 0.035; w, *F*_(2,18)_ = 10.50, *P* < 0.001). GS activity was restored in the STR-YQ group without a change in the expression level (Fig. 4x and y; x, *F*_(2,18)_ = 20.23, *P* < 0.001), and Tyr-nitration of GS was also significantly decreased in the STR-YQ group (Fig. 4z and aa; z, *F*_(2,18)_ = 3.837, *P* = 0.041).

### Tyr and YQ reduce kainic acid-induced epileptic seizures and decrease Tyr-nitration of GS in the hippocampus

**Fig. 5:**
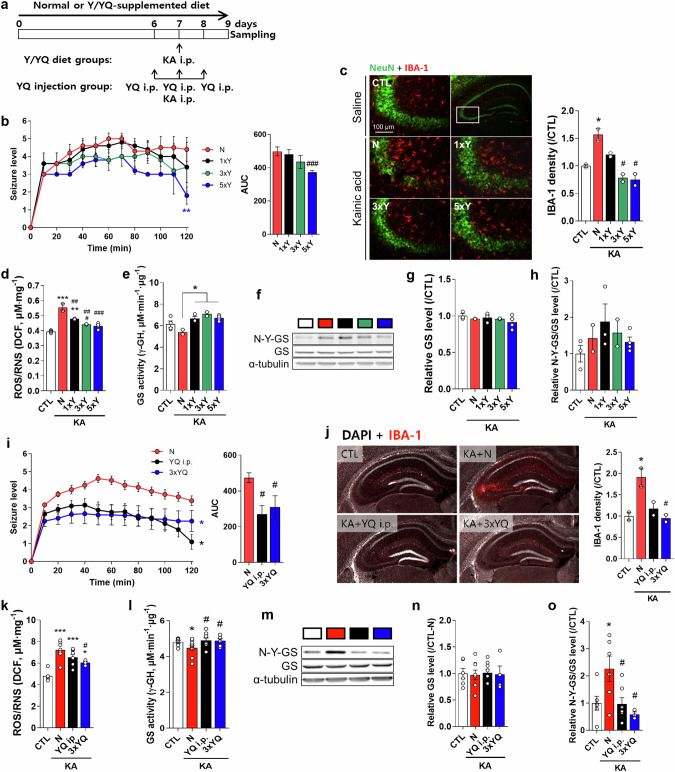
Tyr-Gln (YQ) attenuates kainic acid (KA)–induced seizures and the decrement in glutamine synthetase (GS) activity in the hippocampus. **a** Scheme for Y/YQ diet supplementation and YQ/KA i.p. injections (*n* = 10 per group). **b** and **i** Changes in seizure levels after KA and different treatments (normal diet without KA treatment group: CTL, normal diet with KA treatment group: N, 1×Y diet with KA treatment group: 1×Y, 3×Y diet with KA treatment group; 3×Y, 5×Y diet with KA treatment group: 5×Y, YQ i.p. administration with KA treatment group: YQ i.p., 3×YQ diet with KA treatment group: 3×YQ). **c** and **j** Representative images and relative densities of IBA-1 expression in the CA3 region of the hippocampus (*n* = 4 per group). **d** and **k** Reactive oxygen species (ROS)/reactive nitrogen species (RNS) levels. **e** and **l** GS activity changes in the hippocampus. **f** and **m** Representative Western blot results for N-Y-GS, GS, and α-tubulin. Changes in GS expression (**g** and **n**) and N-Y-GS levels (**h** and **o**) among groups. Data are presented as the mean ± SEM. *P < 0.05, **P < 0.01, ***P < 0.001 vs. CTL and ^#^*P* < 0.05, ^##^*P* < 0.01, ^###^*P* < 0.001 vs. N (one-way ANOVA with multiple comparisons test).

We determined whether Tyr and/or YQ induce GS denitration and reduce seizures caused by kainic acid (KA) (Fig. 5). Tyr and YQ were provided to mice via their diet or intraperitoneal (i.p.) injection (Fig. 5a). Diet supplementation of 5×Y/3×YQ and i.p. administration of YQ (100 mg/kg) decreased seizure levels compared with those in the N group (Fig. 5b and i; b, *F*_(3,44)_ = 3.939, *P* = 0.0142; i, *F*_(2,33)_ = 4.966, *P* = 0.013). KA markedly increased IBA-1 in the CA3 hippocampal region in the KA-N group, which was decreased in the 3×Y, 5×Y, and 3×YQ diet–supplemented groups (Fig. 5c and j; c, *F*_(4,5)_ = 19.95, *P* = 0.003; j, *F*_(3,4)_ = 9.288, *P* = 0.028). ROS/RNS were also increased in hippocampal tissue after KA injection; however, diet supplementation of 3×Y or 5×Y or YQ i.p., 3×YQ, or i.p. injection of YQ reduced ROS/RNS levels (Fig. 5d and k; d, *F*_(4,9)_ = 33.42, *P* < 0.001; k, *F*_(3,21)_ = 14.52, *P* < 0.001). GS activity was reduced by KA, but Tyr or YQ treatment increased GS activity without a change in its expression (Fig. 5e–g, and l–n; e, *F*_(4,10)_ = 5.717, *P* = 0.012; i, *F*_(3,31)_ = 3.278, *P* = 0.034, *t* = 2.358, df = 19, *P* = 0.029, KA-N vs. KA YQ i.p. / *t* = 2.155, df = 16, *P* = 0.047, KA-N vs. KA 3xYQ). GS nitration was also increased by KA but was decreased by Tyr or YQ treatment (Fig. 5f, h, m, and o; o, *F*_(3,18)_ = 4.707, *P* = 0.014, *t* = 2.618, df = 11, *P* = 0.024, KA-N vs. KA YQ i.p. / *t* = 2.450, df = 7, *P* = 0.044, KA-N vs. KA 3xYQ).

### Tyr and YQ alleviate blood ammonia levels and liver dysfunction in hyperammonemia models

**Fig. 6:**
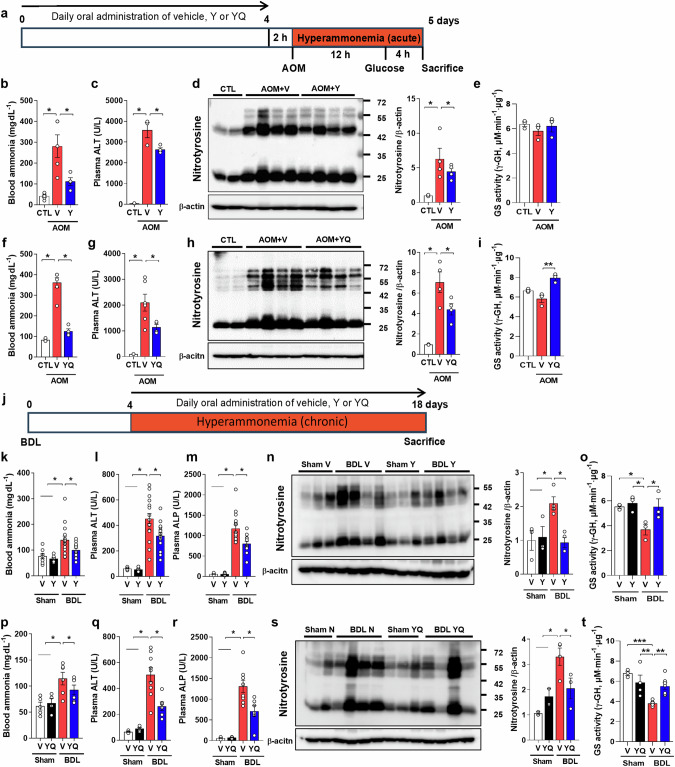
Tyr and Tyr-Gln (YQ) reduce blood ammonia levels and liver injury in azoxymethane (AOM) and bile duct ligation (BDL)–induced hyperammonemia mouse models. **a** and **j** Schemes for Y or YQ administration during AOM and BDL-induced hyperammonemia model, respectively. **b**, **f**, **k**, and **p** Changes in blood ammonia level among groups (normal control: CTL; vehicle administration with AOM, sham, or BDL treatment: V; Y administration with AOM, sham, or BDL treatment: Y; YQ administration with AOM, sham, or BDL treatment: YQ). **c**, **g**, **l**, and **q** Plasma alanine aminotransferase (ALT). **d**, **h**, **n**, and **s** Changes in nitrotyrosine-containing protein levels in the liver. Nitrotyrosine-containing protein levels were normalized to those of β-actin. **e**, **i**, **o**, and **t** GS activity changes in the liver. **m** and **r** Plasma alkaline phosphatase (ALP). Data are presented as the mean ± SEM. *P < 0.05, **P < 0.01, ***P< 0.001 vs. CTL or V groups (multiple comparisons test).

We determined whether Tyr or YQ has a beneficial effect by regulating blood ammonia via GS activation in the liver in two hyperammonemia mouse models, azoxymethane (AOM)-induced liver failure as an acute model and bile duct ligation (BDL)-induced liver failure as a chronic model. In the AOM model, blood ammonia and Tyr-nitration levels in the liver were increased by AOM, but these increments were attenuated by Tyr (100 mg/kg) or YQ (200 mg/kg) treatment (Fig. 6b, d, f, and h; b, *F*_(2,13)_ = 17.42, *P* < 0.001; d, *F*_(2,7)_ = 4.109, *P* = 0.041; f, *F*_(2,10)_ = 54.15, *P* < 0.001; h, *F*_(2,7)_ = 11.08, *P* = 0.007). The elevated plasma ALT level was reduced by Tyr or YQ treatment, implying that Tyr and YQ at least partly protect liver function against AOM toxicity (Fig. 6c and g; c, *F*_(2,10)_ = 170.4, *P* < 0.001; g, *F*_(2,10)_ = 12.26, *P* = 0.002). The decrease in the blood ammonia level may have been due to the maintenance of GS activity by YQ treatment (Fig. 6i. *F*_(2,6)_ = 16.41, *P* = 0.004). Similar to AOM-induced liver failure, BDL-induced liver dysfunction was evidenced by increased blood ammonia and plasma ALT and alkaline phosphatase levels, but these changes were alleviated by Tyr or YQ treatment (Fig. 6k, l, m, p, q, and r; k, *F*_(3,42)_ = 8.448, *P* < 0.001; l, *F*_(3,47)_ = 37.74, *P* < 0.001; m, *F*_(3,43)_ = 64.22, *P* < 0.001; *p*, *F*_(3,17)_ = 5.806, *P* = 0.006; q, *F*_(3,20)_ = 21.93, *P* < 0.001; r, *F*_(3,16)_ = 9.749, *P* < 0.001). Additionally, there was a large increment in Tyr-nitration of liver proteins and a decrement of GS activity by BDL. Chronic oral administration of Tyr (100 mg/kg) or YQ (200 mg/kg) after BDL increased Tyr-denitration and GS activity (Fig. 6n, o, s, and t; n, *F*_(3,10)_ = 6.510, *P* = 0.01; o, *F*_(3,8)_ = 5.099, *P* = 0.029; s, *F*_(3,7)_ = 7.101, *P* = 0.016; t, *F*_(3,16)_ = 9.749, *P* < 0.001).

## Discussion

Tyr-nitration can cause critical structural and functional alterations of proteins, resulting in devastating effects on cell and tissue homeostasis. Tyr-nitrated proteins have not only been found in various disease conditions but are also associated with the aging process [35]. Not all proteins with Tyr residues are nitrated, but particular conditions are associated with preferential protein targets and the extent of nitration. Although we did not identify other CIS-induced Tyr-nitrated proteins in the mPFC, GS activity was obviously decreased when Tyr-nitration was increased by CIS. Therefore, GS may be a preferentially nitrated protein in the mPFC during chronic stress. Interestingly, earlier research suggested that Tyr-nitration of GS is reversible by endogenous putative denitrase [29] or environmental pH changes [36]. We also found that spinach extracts had antistress and antidepressive effects, but these effects were not found for an extract that did not have any Tyr as a free amino acid [37]. Because Tyr in proteins is a target for nitration, we hypothesized that exogenous free Tyr could prevent Tyr-nitration in proteins or have denitrative effects as a nitrite scavenger. Expectedly, Tyr protected GS activity from PN in a dose-dependent manner in rhGS and mouse PFC lysate, which might be due to putative denitrase activity in the brain lysate. It was also confirmed in the CIS-depression mouse model that Tyr-nitration of GS was noticeably decreased, and GS activity was significantly increased without a change in its expression by exogenous Tyr supplementation. Moreover, Tyr showed antistress and antidepressive properties.

The present study is not the first to examine the antidepressive effect of Tyr, because previous studies sought to determine whether Tyr has antistress or antidepressive properties in animal models and double-blind clinical trials [38, 39]. These previous studies investigated whether exogenous Tyr elevates dopamine levels in the brain and has antidepressive effects. Toward this end, a previous study used a Tyr dose of 100 mg·kg^−1^·d^−1^ for 4 weeks and observed elevated dopamine levels in the brain, but it did not find an antidepressive effect [38]. Previous studies were based on the monoamine hypothesis, which has been replaced by the newer, glutamatergic hypothesis [18, 40]. The dose administered to mice in the present study is equivalent to ~1.81 mg·kg^−1^·d^−1^ for humans, which is much less than that used in previous clinical trials. Although we did not analyze brain dopamine or norepinephrine content, the amount administered in our study is not sufficient to increase brain catecholamines [41, 42]. Therefore, the mechanism by which Tyr works in the brain in the present study is different from that in previous studies. The new working mechanism of Tyr suggested by this study includes activation of GS, in other words, enzyme activation, which is a first-in-class strategy for drug development.

Although Tyr shows an antidepressive effect, it is a single amino acid that has several weaknesses for use as a medication, including its stability and solubility [43]. Many trials have used dipeptides containing a useful single amino acid for medication or a medical supplement. Tyr or Tyr- or Gln-containing dipeptides are already being clinically used to help patients with kidney or liver disease or to make infant formula [44–47]. Gln also had antistress and antidepressive properties in a CIS-induced mouse model [16, 37, 48]. Therefore, we decided to make dipeptides consisting of Tyr and Gln. We synthesized and examined the availability of YQ and QY. Tyr-Gln (YQ) and QY showed good solubility in water at ambient temperatures. As expected, the water solubility of YQ was at least 72 g/L, and it showed remarkable stability in citrate buffer at different pH levels, including 4, 6, and 8, at an ambient temperature for 28 days. Moreover, YQ showed a stable reaction curve with a high concentration in an in vitro GS denitration test as well as antidepressive effects and GS activation. However, QY is more difficult to synthesize, thus most other experiments have been performed only with YQ. YQ had an advantage as a dipeptide with high solubility because YQ solution can be prepared for i.p. injection at a concentration of 15 mg/ml, but a similar solution could not be prepared using Tyr because its solubility is 0.49 mg/ml. Therefore, YQ has a more prominent effect on the denitration of GS during epileptic seizures. An advantage of YQ was also found in an AOM-induced acute liver failure model, in which protein denitration and GS activity changes were more prominent in the YQ treatment group than in the Tyr treatment group.

Activation of KA receptors accelerates neuronal depolarization and may result in excitotoxic neuronal injury similar to that of temporal lobe epilepsy (TLE) [49]. Surgical examination of patients with mesial TLE revealed approximately 40% loss of GS protein and its activity in the sclerotic hippocampus. However, loss of GS was not found in surgically resected hippocampi from diseased individuals with no history of epilepsy [50, 51]. Therefore, it is important to maintain GS activity during epilepsy and after seizures to save neurons because ROS/RNS production can be markedly increased by seizure events. In the case of intractable epilepsy in children, because there is no suitable medication, the treatment approach is to undergo surgery or ingest a special diet, such as a ketogenic diet containing high fat and low carbohydrate levels [52]. Thus, a new, safe, and effective medication is urgently needed for children with intractable epilepsy. Tyr or YQ may be a good choice to develop this new medication.

Apart from the brain, GS plays an important role in removing ammonia and keeping the body free from nitrogen waste in the liver [53]. Ammonia is removed by two main pathways, consisting of the urea cycle and GS. If one of these pathways is impaired, an organism might suffer from hyperammonemia. Thus, there have been many attempts to overexpress or restore liver-specific GS expression to treat hyperammonemia because excess ammonia freely crosses the blood-brain barrier and disrupts neuronal homeostasis, leading to brain edema, convulsions, and coma [54]. AOM injection results in acute liver failure with a rapid increment in ROS/RNS, and BDL causes biliary cirrhosis induced by ROS/RNS derived from bile duct obstruction [55–57]. We found that a large amount of protein Tyr-nitration was induced by AOM and BDL in liver tissues, but Tyr or YQ treatment reduced the blood ammonia level and partly restored liver function. GS activity was restored by Tyr or YQ treatment, and thus Tyr or YQ might be a candidate for treatment of acute or chronic liver dysfunction associated with hyperammonemia preceding hepatic encephalopathy.

In the present study, we found a decrement in GS activity during chronic stress in the mPFC that was induced by Tyr-nitration, which led to chronic stress-induced depressive behaviors in mice. Because Tyr-nitration of GS is reversible, we hypothesized that Tyr or Tyr-containing dipeptides could prevent nitration or facilitate denitration of GS during CIS. Consequently, Tyr and YQ showed an antidepressive effect in a CIS-induced mouse depression model, which was accompanied by increased GS activity and reduced Tyr-nitration. The use of acute KA-induced seizure and acute/chronic liver failure models confirmed that Tyr or YQ could be part of a new drug development process to overcome GS dysfunction and related diseases.

## Supplementary information

Supplementary Figure

Supplementary tables

Supplementary methods
