# Supplementary material for: Activation of glutamine synthetase (GS) as a new strategy for the treatment of major depressive disorder and other GS-related diseases
Source: Acta Pharmacol Sin. 2025 Jan 7;46(4):880–91. doi: 10.1038/s41401-024-01441-2 (PMC11950325; doi:10.1038/s41401-024-01441-2)
Supplement: Supplementary file 1 — Supplementary Figure [file 41401_2024_1441_MOESM1_ESM.docx]

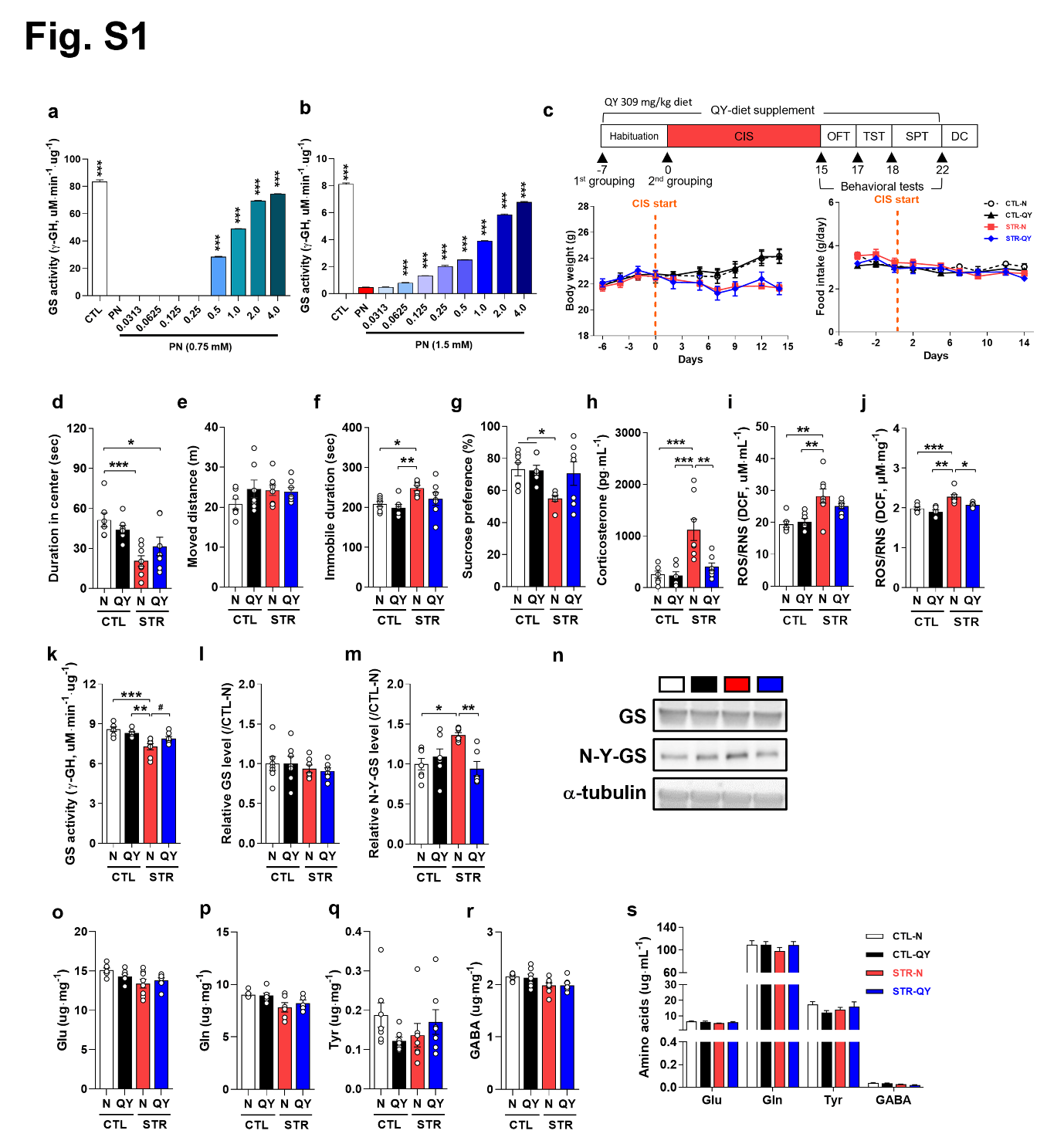


Fig. S1. Gln-Tyr (QY) shows antidepressive effects in chronic immobilization stress (CIS)– induced depressive mice via activation of glutamine synthetase (GS) in the medial prefrontal cortex (mPFC). (a and b) Denitrative effect of QY on human recombinant GS and mouse mPFC lysate. (c) Scheme for QY diet supplementation, CIS, and behavioral tests. Changes in body weight and food intake during the experiment among groups (normal diet control group: CTL-N, QY diet control group: CTL-QY, normal diet stress group: STR-N, QY diet stress group: STR-QY, n=7 per group). (d–g) Behavioral test results: open field test (d and e), tail suspension test (f), and sucrose preference test (g). (h) Plasma corticosterone level. (i and j) Reactive oxygen species (ROS)/reactive nitrogen species (RNS) levels in plasma and the mPFC, respectively. (k and l) Activity and expression levels of GS. (m) N-Y-GS level in the mPFC. (n) Representative western blot images for GS, N-Y-GS, and α -tubulin. (o–r) Glutamate (Glu), glutamine (Gln), Tyr, and γ-aminobutyric acid (GABA) levels in the mPFC. (s) Amino acid (Glu, Gln, Tyr, and GABA) levels in plasma. Data are presented as the mean±SEM. ^*^p<0.05, ^**^p<0.01, ^***^p<0.001 (multiple comparisons test), and ^#^p<0.05 (individual comparison test) vs. CTL-N or STR-N groups.
