# Supplementary material for: Activation of glutamine synthetase (GS) as a new strategy for the treatment of major depressive disorder and other GS-related diseases
Source: Acta Pharmacol Sin. 2025 Jan 7;46(4):880–91. doi: 10.1038/s41401-024-01441-2 (PMC11950325; doi:10.1038/s41401-024-01441-2)
Supplement: Supplementary file 2 — Supplementary tables [file 41401_2024_1441_MOESM2_ESM.docx]

**Supplemental Tables**

**Table S1. Institutional Animal Care and Use Committee (IACUC) numbers for this study.**

| **IACUC number** | **Experiments** |
| --- | --- |
| GNU-190819-M0039 | Depression and mild cognitive impairment induced by chronic immobilization stress |
| GNU-211117-M0099 | Seizures induced by kainic acid |
| GNU-210901-M0075 | Hyperammonemia induced by bile duct ligation |
| GNU-210901-M0076 | Hyperammonemia induced by azoxymethane |

**Table S2. Antibodies used for western blotting and immunohistochemistry.**

| **Name** | **Host** | **Manufacturer** | **Cat. No.** | **Experiment** |
| --- | --- | --- | --- | --- |
| Nitrotyrosine | Mouse | Abcam | ab61392 | WB |
|  | Mouse | Santa Cruz | sc-32757 | IP |
| GS | Mouse | Millipore | MAB302 | WB |
| NeuN | Mouse | Millipore | MAB377 | IHC |
| IBA-1 | Rabbit | Wako | 019-19741 | IHC |
| β-actin | Mouse | Sigma | A5441-2ML | WB |
| α-tubulin | Mouse | ThermoFisher | 32-2600 | WB |

WB, western blotting; IP, immunoprecipitation; IHC, immunohistochemistry
