# Supplementary material for: Activation of glutamine synthetase (GS) as a new strategy for the treatment of major depressive disorder and other GS-related diseases
Source: Acta Pharmacol Sin. 2025 Jan 7;46(4):880–91. doi: 10.1038/s41401-024-01441-2 (PMC11950325; doi:10.1038/s41401-024-01441-2)
Supplement: Supplementary file 3 — Supplementary methods [file 41401_2024_1441_MOESM3_ESM.docx]

**Supplemental methods**

**1. Preparation of animal models of disease**

**1.1. Depression induction by chronic immobilization stress**

Seven-week-old male C57BL/6 mice were individually housed for 7 days before the experiment for acclimatization. For chronic immobilization stress-induced depression, mice were individually forced into a restrainer for 2 h every day for 15 days as previously described (Son et al., 2018).

**1.2. Seizure induction by kainic acid injection**

Four-week-old male ICR mice were fed 1×, 3×, or 5× Y- or 3× YQ-supplemented diets for 1 week. Kainic acid (KA; ab120100, Abcam, Cambridge, UK) was administered via intraperitoneal (i.p.) injection into 5-week-old mice (30±3 g) at a dose of 39 mg/kg body weight. For YQ injection, YQ solution was injected (i.p., 100 mg/kg, 3 mg YQ/200 μl water/30 g body weight) three times (24 and 1 h before and 24 h after KA injection) into normal diet-fed 5-week-old mice. Seizure levels were assessed according to a previous study (Morrison et al., 1996) and were recorded for 2 h. Mice were sacrificed 2 days after KA injection.

**1.3. Acute liver injury and hyperammonemia**

Thirteen-week-old male C57BL/6 mice were habituated for 1 week, after which acute liver failure was induced by a single i.p. injection of azoxymethane (AOM; A5486, Merck, Rahway, NJ, USA; 100 mg/kg) or saline as a control. The mice injected with AOM were orally administered carboxymethyl cellulose (CMC; Sigma-Aldrich) vehicle, Y (100 mg/kg), or YQ (200 mg/kg) once a day for 4 days prior to AOM injection. After 12 h, mice were injected with 5% glucose in 200 µl saline to reduce hypoglycemia and dehydration. Bile duct ligation (BDL) was conducted as described in previous studies under sterile conditions (Cho et al., 2020; Claeys et al., 2022). BDL mice were orally administered CMC vehicle, Y (100 mg/kg), or YQ (200 mg/kg). After 14 days, BDL mice were sacrificed.

**2. Biochemical and molecular approaches**

**2.1. Measurements of ammonia, alanine aminotransferase, alkaline phosphatase, corticosterone, and reactive oxygen species/reactive nitrogen species**

Plasma alanine aminotransferase and alkaline phosphatase levels were measured using commercial assay kits from IVD Lab (Uiwang, Rep. of Korea). Corticosterone (CORT) and reactive oxygen species (ROS)/reactive nitrogen species (RNS) were quantified using a Corticosterone ELISA Kit (501320, Cayman Chemicals, Ann Arbor, MI, USA) and an OxiSelect™ In Vitro ROS/RNS Assay Kit (STA-347, Cell Biolabs, San Diego, CA, USA) according to protocols provided by the manufacturers. CORT and ROS/RNS were measured in plasma and brain tissues, respectively.

**2.2. Glutamine synthetase activity assay**

Glutamine synthetase (GS) activity was measured as described in a previous study (Peng et al., 2016). The lysate or recombinant human GS (rhGS; NBP2-52619, Novus Biologicals, Centennial, CO, USA) diluted in GS lysis buffer (50 mM imidazole, pH 6.8) was mixed with GS assay buffer (50 mM imidazole-HCl, pH 6.8, 50 mM L-glutamine, 25 mM hydroxylamine, 25 mM sodium arsenate, 2 mM MnCl_2_, and 0.16 mM ADP) and incubated for 1 h at 37 °C. After adding the stop solution (90 mM FeCl_3_, 1.8 N HCl, and 1.45% trichloroacetic acid), the absorbance was measured at 560 nm using a VersaMax microplate reader (Molecular Devices, San Jose, CA, USA). GS activity was calculated as the amount of γ-glutamylhydroxamate produced per minute by the amount of lysate or rhGS in the reaction. All chemicals were purchased from Sigma-Aldrich, and information regarding the chemicals was included in a previous study (Peng et al. 2016).

**2.3. Western blotting**

Denatured lysates were separated by sodium dodecyl sulfate-polyacrylamide gel electrophoresis and transferred to polyvinylidene difluoride membranes (1704157, Bio-Rad, Hercules, CA, USA). The membranes were blocked in 5% skim milk diluted in tris-buffered saline and 0.1% Tween 20 and sequentially incubated with primary and appropriate secondary antibodies. Information on the antibodies used in this study is provided in Table S2. Images were detected using an iBright™ CL1500 Image System (ThermoFisher Scientific) or ChemiDoc XRS+ System (Bio-Rad). Relative protein levels were quantified using iBright Analysis Software (ThermoFisher Scientific) or Image Lab^TM^ software (Bio-Rad).

**2.4. Immunoprecipitation**

Immunoprecipitation of nitrotyrosine was conducted using an anti-3-NT antibody (sc-32757, Santa Cruz, Dallas, TX, USA) and Protein A/G Plus Agarose (sc-2003, Santa Cruz) according to the protocol provided by the manufacturer.

**2.5. Immunochemistry**

Mice were deeply anesthetized with avertin and perfused with phosphate-buffered saline (PBS, pH 7.4) and 4% (wt/vol) paraformaldehyde in PBS. The brains were collected, post-fixed, and sectioned at 40-μm thickness using a Leica VT1200 vibratome (Leica, Wetzlar, Germany). Brain sections were incubated at 4 °C overnight with anti-neuronal nuclei (NeuN) (MAB377, Merck Millipore) and ionized calcium-binding adapter molecule 1 (IBA1) (019-19741, Wako, Osaka, Japan) antibodies. The slices were then incubated with Alexa Fluor 594- and/or 488-conjugated secondary antibodies (1:200, Invitrogen). Digital images were captured using a spinning disk confocal microscope (Olympus, Tokyo, Japan) and analyzed with ImageJ software (NIH).

**References**

Cho, I., Koo, B.N., Kam, E.H., Lee, S.K., Oh, H., Kim, S.Y., 2020. Bile duct ligation of C57BL/6 mice as a model of hepatic encephalopathy. Anesth Pain Med (Seoul) 15 (1), 19-27.

Claeys, W., Van Hoecke, L., Geerts, A., Van Vlierberghe, H., Lefere, S., Van Imschoot, G., Van Wonterghem, E., Ghesquiere, B., Vandenbroucke, R.E., Van Steenkiste, C., 2022. A mouse model of hepatic encephalopathy: bile duct ligation induces brain ammonia overload, glial cell activation and neuroinflammation. Sci Rep 12 (1), 17558.

Morrison, R.S., Wenzel, H.J., Kinoshita, Y., Robbins, C.A., Donehower, L.A., Schwartzkroin, P.A., 1996. Loss of the p53 tumor suppressor gene protects neurons from kainate-induced cell death. J Neurosci 16 (4), 1337-1345.

Peng, I.C., Bott, A.J., Zong, W.X., 2016. Spectrophotometric Determination of Glutamine Synthetase Activity in Cultured Cells. Bio Protoc 6 (19).

Son, H., Baek, J.H., Go, B.S., Jung, D.H., Sontakke, S.B., Chung, H.J., Lee, D.H., Roh, G.S., Kang, S.S., Cho, G.J., Choi, W.S., Lee, D.K., Kim, H.J., 2018. Glutamine has antidepressive effects through increments of glutamate and glutamine levels and glutamatergic activity in the medial prefrontal cortex. Neuropharmacology 143, 143-152.
